# Influence of Being Embodied in an Obese Virtual Body on Shopping Behavior and Products Perception in VR

**DOI:** 10.3389/frobt.2018.00113

**Published:** 2018-10-03

**Authors:** Adrien Verhulst, Jean-Marie Normand, Cindy Lombart, Maki Sugimoto, Guillaume Moreau

**Affiliations:** ^1^CRENAU, AAU UMR CNRS 1563, Computer Science and Mathematics Department, École Centrale de Nantes, Nantes, France; ^2^Hybrid, Inria, Rennes, France; ^3^In Situ Lab, Marketing Department, Audencia Business School, Nantes, France; ^4^Interactive Media Lab, Department of Information and Computer Science, Faculty of Science and Technology, Keio University, Kanagawa, Japan

**Keywords:** virtual reality, virtual embodiment, presence, stereotype activation, user studies, food perception, purchase behavior

## Abstract

Research in Virtual Reality (VR) showed that embodiment can influence participants' perceptions and behavior when embodied in a different yet plausible virtual body. In this paper, we study the changes an obese virtual body has on products perception (e.g., taste, etc.) and purchase behavior (e.g., number purchased) in an immersive virtual retail store. Participants (of a normal BMI on average) were embodied in a normal (N) or an obese (OB) virtual body and were asked to buy and evaluate food products in the immersive virtual store. Based on stereotypes that are classically associated with obese people, we expected that the group embodied in obese avatars would show a more unhealthy diet, (i.e., buy more food products and also buy more products with high energy intake, or saturated fat) and would rate unhealthy food as being tastier and healthier than participants embodied in “normal weight” avatars. Our participants also rated the perception of their virtual body: the OB group perceived their virtual body as significantly heavier and older. They also rated their sense of embodiment and presence within the immersive virtual store. These measures did not show any significant difference between groups. Finally, we asked them to rate different food products in terms of tastiness, healthiness, sustainability and price. The only difference we noticed is that participants embodied in an obese avatar (OB group) rated the *coke* as being significantly tastier and the apple as being significantly healthier. Nevertheless, while we hypothesized that participants embodied in a virtual body with obesity would show differences in their shopping patterns (e.g., more “unhealthy” products bought) there were no significant differences between the groups. Stereotype activation failed for our participants embodied in obese avatars, who did not exhibit a shopping behavior following the (negative) stereotypes related to obese people. conversely, while the opposite hypothesis (participants embodied in obese avatars would buy significantly more healthy products in order to “transform” their virtual bodies) could have been made, it was not the case either. We discuss these results and propose hypotheses as to why the behavior of the manipulated group differed from the one we expected. Indeed, unlike previous research, our participants were embodied in virtual avatars which differed greatly from their real bodies. Obese avatars should not only modify users' visual characteristics such as hair or skin color, etc. We hypothesize that an obese virtual body may require some other non-visual stimulus, e.g., the sensation of the extra weight or the change in body size. This main difference could then explain why we did not notice any important modification on participants' behavior and perceptions of food products. We also hypothesize that the absence of stereotype activation and thus of statistical difference between our N and OB groups might be due to higher-level cognitive processes involved while purchasing food products. Indeed our participants might have rejected their virtual bodies when performing the shopping task, while the embodiment and presence ratings did not show significant differences, and purchased products based on their real (non-obese) bodies. This could mean that stereotype activation is more complex that previously thought.

## 1. Introduction

### 1.1. Context

Virtual retail stores are becoming a usual tool to conduct consumer behavioral studies. There are several advantages of using a virtual store (with immersive VR technologies or 3D desktop-based only) over a physical one, such as cost and time efficiency, control of experimental conditions, scalability, see Breen (2009). Overall, virtual stores present comparable but slightly different buying processes (e.g., shopping patterns, proportional purchases within food group, etc.) from physical ones, cf. Waterlander et al. (2015). Indeed, it has been shown that consumers tend to buy more products in a virtual store (Burke et al., 1992), spend more money (List and Gallet, 2001), or purchase a larger variety of products, see van Herpen et al. (2016).

In this research, we introduce a difference between *immersive virtual stores* and *3D desktop-based* virtual stores. We call an *immersive virtual store*, a virtual store using immersive virtual reality techniques. Obviously, among immersive virtual stores, there are different degrees of immersion, such as those with Head Mounted Display (HMD) and *only* head-tracking (i.e., commonly called immersive virtual stores), and those with HMD and full-body tracking (cf. Spanlang et al., 2014). On the other hand, we call *3D desktop-based* a virtual store using 3D computer graphics techniques (e.g., a game-like environment displayed on a desktop computer and for which user interaction relies on a keyboard and a computer mouse).

Full body tracking allows to replicate movements of the participant's real body on his/her virtual body (usually called the avatar). This can induce an “illusion” in which the participant associates his/her virtual body as his/her real body, which is called virtual *embodiment* or virtual Body Ownership Illusions (BOI)s (cf. section 2.1). Studies showed that the virtual body has an impact on a participant's perception and behavior (see e.g., Kilteni et al., 2013; Slater and Sanchez-Vives, 2014). More specifically, the participant adapts his/her perception and behavior to conform to what the participant expects his/her virtual body to perceive/react to. This raises the question: could the virtual body alone influence participants' consumer behavior?

Embodiment has been shown to elicit changes in perceptions and behavior. The notion of *body semantics*, Slater and Sanchez-Vives (2014), also called *stereotype activation*, suggests that people tend to behave according to their bodies' stereotypes or to their previous experience of how people with the same body type behaved.

The objective of our study is to determine whether a virtual body exhibiting an unhealthy diet (i.e., an obese body) could elicit changes in participants' products perception and consumer purchase behavior in a virtual retail store. In order to focus on the sole influence of the virtual body, we did not include social or environmental cues in the virtual store (such as other customers or food labels indicating nutritional information). In order to do so, we designed an experiment where participants were embodied either in an **obese virtual avatar** (group **OB**) or in a “**normal**”, according to the World Health Organization (WHO)'s Body Mass Index (BMI) classification (see more details below) virtual avatar (group **N**).

### 1.2. Research hypotheses

There exist several reasons to focus on a body exhibiting stereotypes of an unhealthy diet:

**A characteristic body type**. Obesity is a medical condition defined by the WHO as having a BMI ≥ 30 *kg*/*m*^2^ (World Health Organization, 2017a). Let us recall that BMI is based on the weight (W) in *kg* and height (H) in *m* and computed as: BMI = *W*/*H*^2^. Previous work showed that bodies with a “normal” BMI (i.e., ∈[18.5;24.9] *kg*/*m*^2^, see World Health Organization, 2017a) were judged as healthy when presented to other people (Brierley et al., 2016). Hence, modifying the BMI of an avatar is very characteristic of the representation of a “non-overweight and healthy” or “overweight and unhealthy” virtual body.

**A universal perception of obesity and *weight stigma***. While this is not the case for all stereotypes, those related to obese people (their behavior, characteristics, etc.) are prevalent and common in most developed countries; the obesity stigma is ubiquitous (see Puhl and Heuer, 2009; Sikorski et al., 2016). There is evidence (World Health Organization, 2017d) that individuals with obesity suffer stigma from educators, employers, health-care professionals, the media and even from family and friends. It has consistently been demonstrated in psychology via experimental research that obese persons are stigmatized because their weight is perceived to be caused by factors within personal control (e.g., overeating and lack of exercise), see more in Puhl and Heuer (2009, 2010).

Indeed, it is generally admitted, that overweight and obesity are associated with widespread strong negative stereotypes, called weight stigma, especially regarding the lack of self-discipline or self-control and overeating (see Gearhardt et al., 2012). Puhl and Heuer (2009) performed a review of articles related to weight bias between 2000 and 2008 and show a shared negative stereotype between overweight/obesity and lack of self-control and overeating. Regarding public views on obesity, (Lee et al., 2013), conducted an online survey (*n* = 479) where 90% of their participants stated that obesity is due to overeating.

It should be reminded that this weight stigma, i.e., the belief that overweight or obesity are primarily die to overeating and a lack of self-control is a belief and not properly represent scientific data (see Crandall, 1994).

However, it should be reminded that obesity is a very complex condition that cannot only be reduced to an unhealthy diet. While it is generally agreed that obesity is a result of an imbalance between the calories consumed and expended (World Health Organization, 2017c), as many other medical conditions, it also depends on environmental and genetics factors (see e.g., Sun et al., 2017). Recently, studies (e.g., Naseer et al., 2014) pointed out the important role of gut microbial environments in relation to obesity and diabetes in human beings and that gut microbiotal environments depend on a complex interplay between ethnicity, genetics, dietary habits and history of medication (Meijnikman et al., 2018). Nevertheless, in our study, we made the assumption that participants would be more familiar with the simplified stereotype connecting in a more direct way obesity and (unhealthy) diet.

**A different perception and behavior regarding products' healthiness**. Products' healthiness is one of the most influent factors in consumers' purchase behavior (see Roininen et al., 1999). Yet, overweight people tend to buy more unhealthy food (with high calories intake), cf. Sturm and An (2014). There are several explanations including SocioEconomic Status (SES) factors (e.g., income, education, etc., see Sobal, 2010) and *weight stigma* (overweight people buy more unhealthy food to conform to their stereotype, cf. Major et al., 2013). Weight is also a known-factor influencing the perception of food's healthiness, and the estimation of calories content (cf. Carels et al., 2006).

Based on these observations, we formulate the following hypothesis:

- **HH1**: Participants embodied in an obese avatar will buy more unhealthy food products (with a high calorie content) and less healthy food products (such as fruits and vegetables) in an immersive virtual store than participants embodied in a “normal” avatar.

**A mis-estimation of food products' caloric content**. Studies such as Larkin and Martin (2016) and Carels et al. (2006, 2007), have shown that people in general (“normal weight,” overweight, obese) usually underestimate the caloric content of healthy foods [obese tend to underestimate it by a greater amount than normal weight or overweight people, see Carels et al. (2006)]. People also tend to overestimate the caloric content of unhealthy food, see Carels et al. (2006, 2007). Moreover, Larkin and Martin (2016) and Carels et al. (2006) showed that obese people are less accurate than normal weight or overweight people.

Tooze et al. (2004) also shown that obesity, quantified by body mass index (BMI; in *kg*/*m*^2^) or percentage of total body fat, is associated with underreporting of energy intake.

Livingstone and Black (2003) also reported that profound underreporting was found in obese subjects and that a negative association between the extent of underreporting and measures of weight status (body weight, percentage body fat or BMI) has also been found in studies that have encompassed a range of body sizes.

Consequently, and based on the *weight stigma* stereotype mentioned above, we posit our second research hypothesis:

- **HH2**: Participants embodied in an obese avatar will perceive food products' healthiness and food products' calorie content as less unhealthy in an immersive virtual store than those embodied in a “normal” avatar.

According to these observations, we hypothesize that an obese body is a good candidate to elicit body semantics from embodied participants: obese people tend to have different product perception and are more prone to “hedonic hunger” (i.e., food consumption driven by pleasure and not only by the need for calories, see Lowe and Butryn, 2007; Cappelleri et al., 2009; Ribeiro et al., 2018), are stigmatized by similar prevalent stereotypes and have a very characteristic body type.

In the remainder of this paper, we present a brief background on embodiment in VR, as well as the *priming effect* (i.e., how environmental cues influence cognition, affect, and behavior) of avatars; we then detail our virtual environment and the experimental protocol before presenting our results and discussing them.

## 2. Background

In this section, we present a review of embodiment in VR.

### 2.1. Virtual embodiment and virtual body illusions

Virtual embodiment is referred to as “the physical process employing VR hardware and software to substitute a person's *real body* with a *virtual body*” (Spanlang et al., 2014). Under certain conditions, it can elicit the subjective Sense Of Embodiment (SoE), commonly defined as “SoE toward a body B is the sense that emerges when B's properties are processed as if they were the properties of one's own biological body” (Kilteni et al., 2012a).

It is composed of three “sensations”:

- **Self-location**: one's spatial experience of being inside a (virtual) body. It is highly determined by visual perspective (Blanke and Metzinger, 2009), as well as by vestibular (Lopez et al., 2008) and sensory stimulations (Lenggenhager et al., 2009).
- **Agency**: the sense of having the subjective experience of action, i.e., “global motor control”. It is the feeling that the person is the agent of its own actions, and is highlighted if there is correspondence between the perceived and the actual consequence of an action (David et al., 2016).
- **Body Ownership**: one's self-attribution of a body. It is the feeling that the virtual body is the source of sensations (Tsakiris et al., 2006) and emerges from a combination of visuotactile and visuoproprioceptive correlations (Blom et al., 2014) as well as morphological similarities (Lugrin et al., 2015).

For further details, we invite the reader to refer to Kilteni et al. (2012a).

#### 2.1.1. Virtual body ownership illusions

Those three sensations are able to elicit BOIs, where the user associates his/her fake body (or fake body parts) with his/her real body. The possibility for people to “quickly learn to inhabit strange and different bodies and still interact with the virtual world” (i.e., the *homoncular flexibility*) was observed by Lanier (2006) with different avatars (humanoid or not) in VR.

In 1998, Botvinick and Cohen (1998) showed with the Rubber Hand Illusion (RHI) that temporally and spatially synchronous sensory stimulations of a fake hand and of the participants' real hand led them to consider the fake hand as their real hand. This BOI is not limited to the hand, and can be elicited between a manikin and a real body as shown by Petkova et al. (2011). The RHI was successfully replicated in VR, (see Yuan and Steed, 2010), and extended to an entire virtual body in Slater et al. (2010). Still in VR, Maselli and Slater (2013) showed that virtual BOIs could be achieved without congruent multisensory cues if the virtual body was realistic enough (e.g., with a convincing skin-tone) and in a similar posture. If the virtual body was not realistic enough, congruent multisensory cues were needed to achieve BOIs. The authors confirmed that first person perspective (i.e., self-location) was necessary.

Several studies have shown that, through virtual BOIs (i.e., with a virtual body or body parts) participants associated their virtual body as their own (Slater et al., 2010; Maselli and Slater, 2013), which is likely to lead to cognitive (cf. Peck et al., 2013; Bergström et al., 2016; perceptual cf. Normand et al., 2011; Kilteni et al., 2012b, or behavioral changes, see Banakou et al., 2013; Kilteni et al., 2013; Slater and Sanchez-Vives, 2014).

Related to our subject of study, we can point out the two following papers: Normand et al. (2011) and Piryankova et al. (2014) that consider embodiment of participants with avatars of considerably different size with respect to their physical bodies.

In Normand et al. (2011), male participants were seated at a table and embodied in a virtual body with a considerable belly. The authors showed that synchronous visuotactile stimulations on the virtual and the real belly induced a perception of a bigger real belly on participants.

In a somewhat similar setup, Piryankova et al. (2014) showed that women can experience ownership of a whole virtual body that is considerably larger or smaller than their real body. Unlike Normand et al. (2011), where the estimation of participants' body size was carried out by the participants themselves in the virtual environment (by adjusting the size of the avatar's belly), in Piryankova et al. (2014) the size estimation was carried out by two different measures: an affordance estimation (users had to adjust the distance between two poles so that they can pass through them) and a body size estimation (users saw a virtual body from a third person perspective and could adjust its size).

Note that in both setups participants were seating at a table and were not moving freely. This is an important difference with our current setup.

#### 2.1.2. Changes of perception and behavior under virtual body ownership illusions

The SoE can elicit both (1) the “social self-perception” concept, where users conform their behavior to how others expect them to behave with that body, (see Yee and Bailenson, 2007) and (2) “self-perception without social cues”, where users conform their behavior to that body, without consideration of others' expectations, see Slater and Sanchez-Vives (2014).

Yee and Bailenson (2007) showed that, in social interactions, an altered “self-representation has a significant and instantaneous impact on [the participant's] behavior,” an effect termed as the *Proteus Effect*. Participants embodied with an attractive face had a shorter interpersonal distance and disclosed more personal information when talking with someone of the opposite sex. Participants with a taller body were shown to be more confident when negotiating.

Kilteni et al. (2013) showed that virtual BOIs can: (1) be induced even if the real and the virtual bodies are from different demographics, confirming results of Groom et al. (2009); (2) elicit behavioral changes which strength is positively correlated with that of the BOIs.

Virtual BOIs can also elicit changes in perception and attitude. Banakou et al. (2013) showed that the virtual body's perceived age could influence the perception of objects' size. Participants embodied in a child virtual body overestimated the size of objects more than those embodied in an adult virtual body of the same height. Peck et al. (2013) observed that a light-skinned participant embodied in a dark-skinned avatar showed a temporary reduced implicit racial bias against dark-skinned avatars after leaving the Virtual Environment (VE). The authors argued that the embodiment transformed momentarily the participant's own group affiliation (i.e., transfer from light-skinned to dark-skinned), which in turn reduced their social bias against the dark-skinned group. Banakou et al. (2016) extended on those results and found that decreased social bias was still visible after a week.

### 2.2. Influence of self-avatars, stereotype activation

Several studies investigated the effect of self-avatars on cognition, emotion and behavior. While priming research focuses on environmental cues (sound, color, etc.), there are obvious similarities between embodiment and the priming effect (or stereotype activation) of self-avatars: both elicit attitude, affect and behavior changes driven only by self-avatars.

Self-avatars have been shown to automatically activate participants' related knowledge. Peña et al. (2009) showed that avatars' appearance can elicit aggressive attitudes: in a 3D desktop-based setup, participants with black-cloaked avatars were more aggressive than those with white cloaks. Using the same setup, Peña et al. (2012) illustrated that avatars' appearance and role (professor or supermodel) impacted users' cognition: participants with a model avatar wrote stories involving brands, exotic names, etc., while participants with a professor avatar wrote stories involving books, education, etc.

Some studies used priming effects in marketing. Bailenson and Ahn (2011) showed that interacting in VR with a product while wearing a promotional shirt of the product led to a more favorable brand attitude and purchase intention. Yoo et al. (2015) studied how differences between avatars' and consumers' age impacted purchase and pro-social behavior. In a 3D desktop-based virtual store, they showed that an elderly avatar made the participant: (1) walk significantly slower in the VE; (2) choose a magazine targeting elderly people more frequently than one targeting young people; (3) give significantly more money to a charitable organization for seniors.

## 3. Virtual environment and materials

We now detail the avatars, the VE and the materials we used.

### 3.1. The avatars

The avatars were procedurally generated (modeled, rigged and skinned) using Blender v2.7 and the ManuelbastioniLAB add-on v1.3^1^ for Blender. Their clothes were manually modeled using Marvelous Designer 6^2^. The avatars were imported into Unity3D v5.5^3^ with a custom-made skin shader for a more realistic appearance.

**Figure 1:**
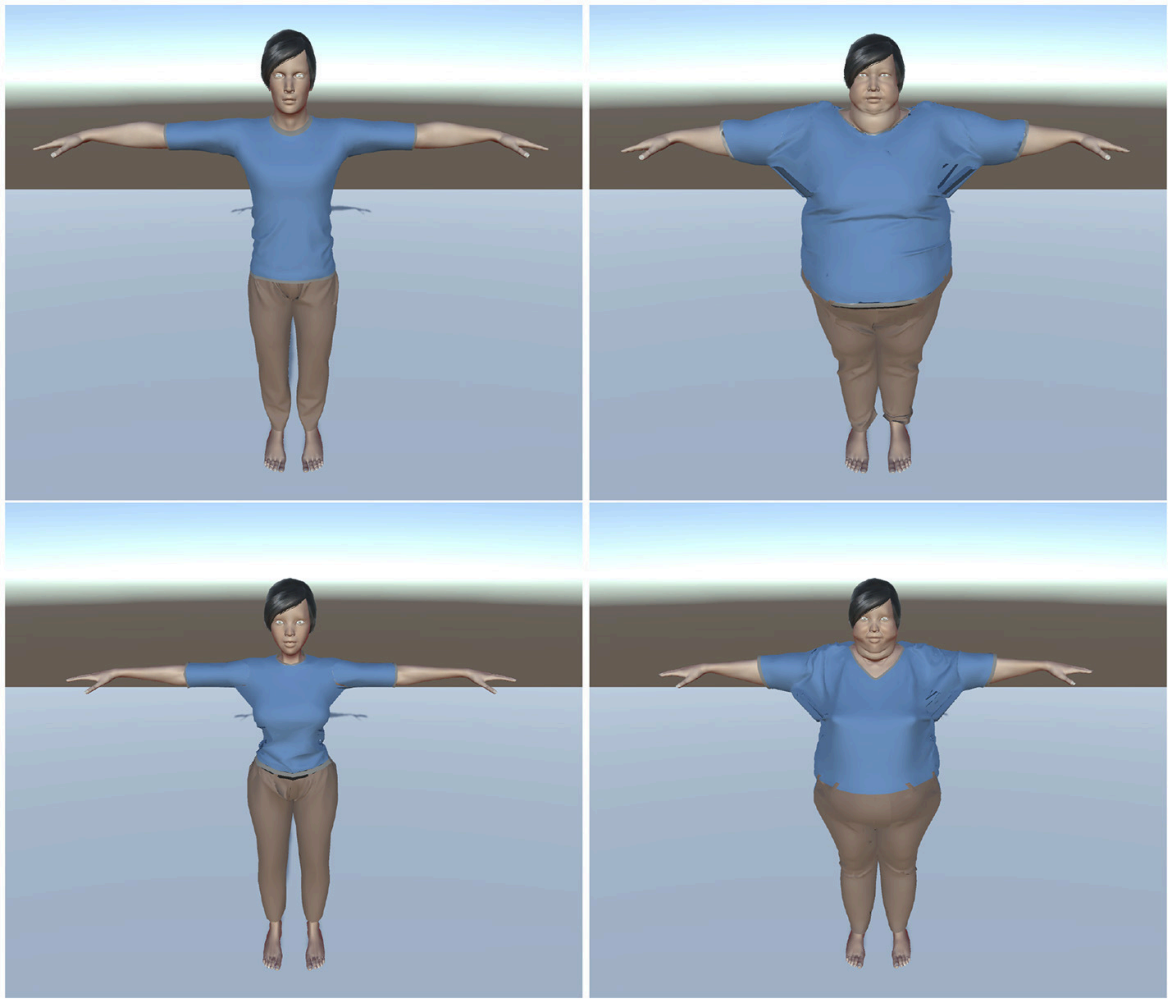
The normal (group N, **left**) and the obese (group OB, **right**) virtual bodies used for the male **(top)** and female **(bottom)** participants.

We had four (2 men and 2 women, cf. Figure 1) fully rigged avatars generated with an *East Asian* preset and a *25 years* preset in ManuelbastioniLAB since most of our participants were *Asian* undergraduate and graduate students (it is worth noting that an avatar's nationality does not have any significant impact on embodiment (see Groom et al., 2009). Moreover, the avatars' height was set to the national Japanese height average (171.5 *cm* for males and 158.5 *cm* for females, cf. Morisaki et al., 2017) and then scaled to meet the participant's real height. In order to compute the avatars' BMI, we estimated the avatars' weight from Velardo's (Velardo and Dugelay, 2010) “weight estimation from visual appearance formula”:

(1) $$\begin{array}{l} W_{\text{est}}=-122.27+0.48\times f_{1}-0.17\times f_{2}+0.52\times f_{3}\\ +0.16\times f_{4}+0.77\times f_{5}+0.49\times f_{6}+0.58\times f_{7} \end{array}$$

where *f*_*i*_ are (in *cm*):

*f*_1_ height;
*f*_2_ upper leg length;
*f*_3_ calf circumference;
*f*_4_ upper arm length;
*f*_5_ upper arm circumference;
*f*_6_ waist circumference;
*f*_7_ upper leg circumference.

The resulting BMI categories (cf. World Health Organization, 2017a) for our avatars were:

- **[Avatars of group N]** Normal BMI (i.e., BMI ≥18.5 and ≤ 25 *kg*/*m*^2^):
  - Male: BMI = 23.25 *kg*/*m*^2^, approximately corresponding to a man of 172 cm weighing ≈ 69 kg.
  - Female: BMI = 19.5 *kg*/*m*^2^, approximately corresponding to a woman of 158 cm weighing ≈ 49.5 kg.
- **[Avatars of group OB]** Obese Class III BMI (i.e., BMI ≥40 *kg*/*m*^2^):
  - Male: BMI ≈ 53 *kg*/*m*^2^, approximately corresponding to a man of 172cm weighing ≈ 157.5 kg.
  - Female: BMI ≈ 49.5 *kg*/*m*^2^, approximately corresponding to a woman of 158.5 cm weighing ≈ 124.2 kg.

It should be noted that while both male and female avatars of the N group are considered as of normal BMI, there is a slight difference in their BMI values: 23.5 *kg*/*m*^2^ for the male avatar and 19.5 *kg*/*m*^2^ for the female avatar. When designing the avatars with the ManuelbastioniLAB add-on we did not only rely on BMI computation but also on the visual aspect of the avatars. As a consequence, a female avatar with a BMI of 23.5 *kg*/*m*^2^ seemed significantly heavier visually than the male avatar.

We propose two explanations for this: the ManuelbastioniLAB add-on might reproduce stereotypes about women representation. This might also be explained by the gender difference in fat metabolism: female tend to have a higher percentage of body fat than men (see Blaak, 2001). Consequently, in order to have a similar visual aspect the female avatar needed to have a smaller BMI index. In the same vein, we noticed a similar difference between male and female avatars of the OB group.

Finally, we chose to use avatars with severe obesity in order to make sure that the participants would notice the difference between their real body and the avatar's body.

### 3.2. The virtual store

We present below the virtual store and the interaction metaphor.

#### 3.2.1. Area and furniture

**Figure 2:**
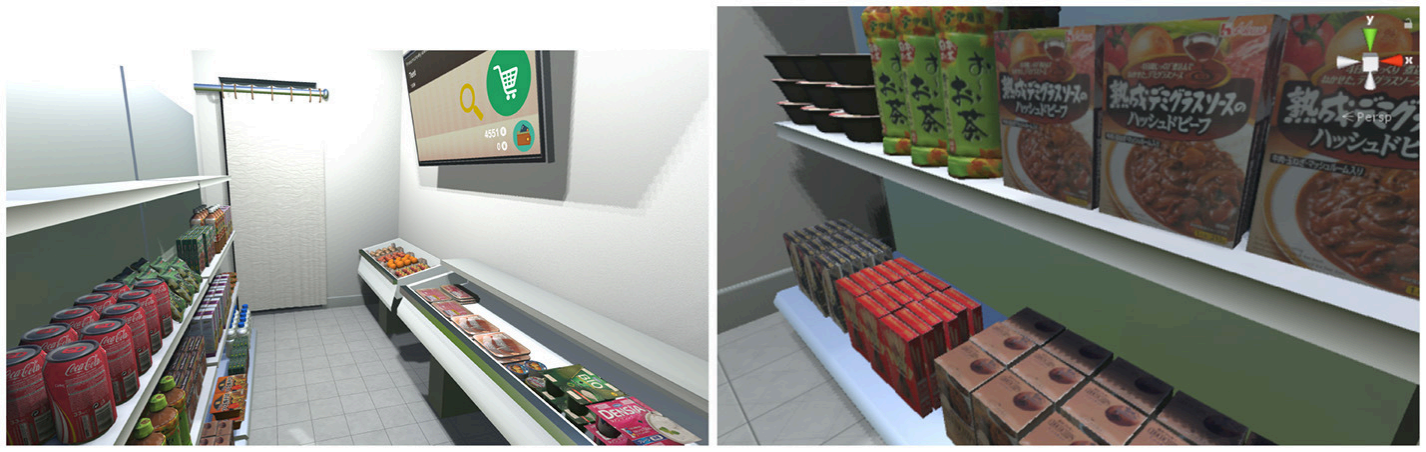
Our virtual store, products are displayed on both sides. **Left:** The TV used for user interaction is attached on a wall. **Right:** A close-up on some products.

The virtual store (including furniture, cf. Figure 2) was manually modeled in Blender v2.7. Since the tracking space was 6*m*^2^ (3 × 2*m*), the store was designed to be of equal size (3.5*m*×1.7 = 5.95*m*^2^) and included:

- 2 shelves for non-fresh products (1.2*m* each);
- 1 stand for fruits and vegetables (1*m*);
- 1 stand for meat and fish (1*m*);
- 1 stand for dairy products (0.65*m*).

It is worth noting that no participant exited the tracking space or tried to walk through the shelves during the experiment, yet they approached the products in order to inspect them.

A large window (1.5 × 1.3*m*) allowed participants to see outside the store (a parking lot and a country-side landscape) and, more importantly, the reflection of their virtual body. A TV was also present, see Figure 2, to display all the information required for the experiment (e.g., selected product, remaining money, questionnaires, etc.) with which interaction was possible via gaze tracking.

#### 3.2.2. Gaze-based interaction metaphor

In order to select a product, participants had to look at it for 0.5 s. Once selected, the product was displayed on the TV and could be added in the basket by looking at the “add to basket” button on the TV (for 1.0 s). Once added in the basket, the product disappeared from the VE. Participants could remove a product from their basket by looking at the “remove from basket” button (also for 1.0 s) and then at the product name (for 2.0 s, the timing was longer to avoid removing a product by mistake). Once removed from the basket, the product reappeared in the VE, where it used to be. The TV also displayed and updated the amount currently spent by participants, as well as the remaining money. During each interaction with the VE, there was a visual and audio feedback in order to facilitate the users' interaction.

**Figure 3:**
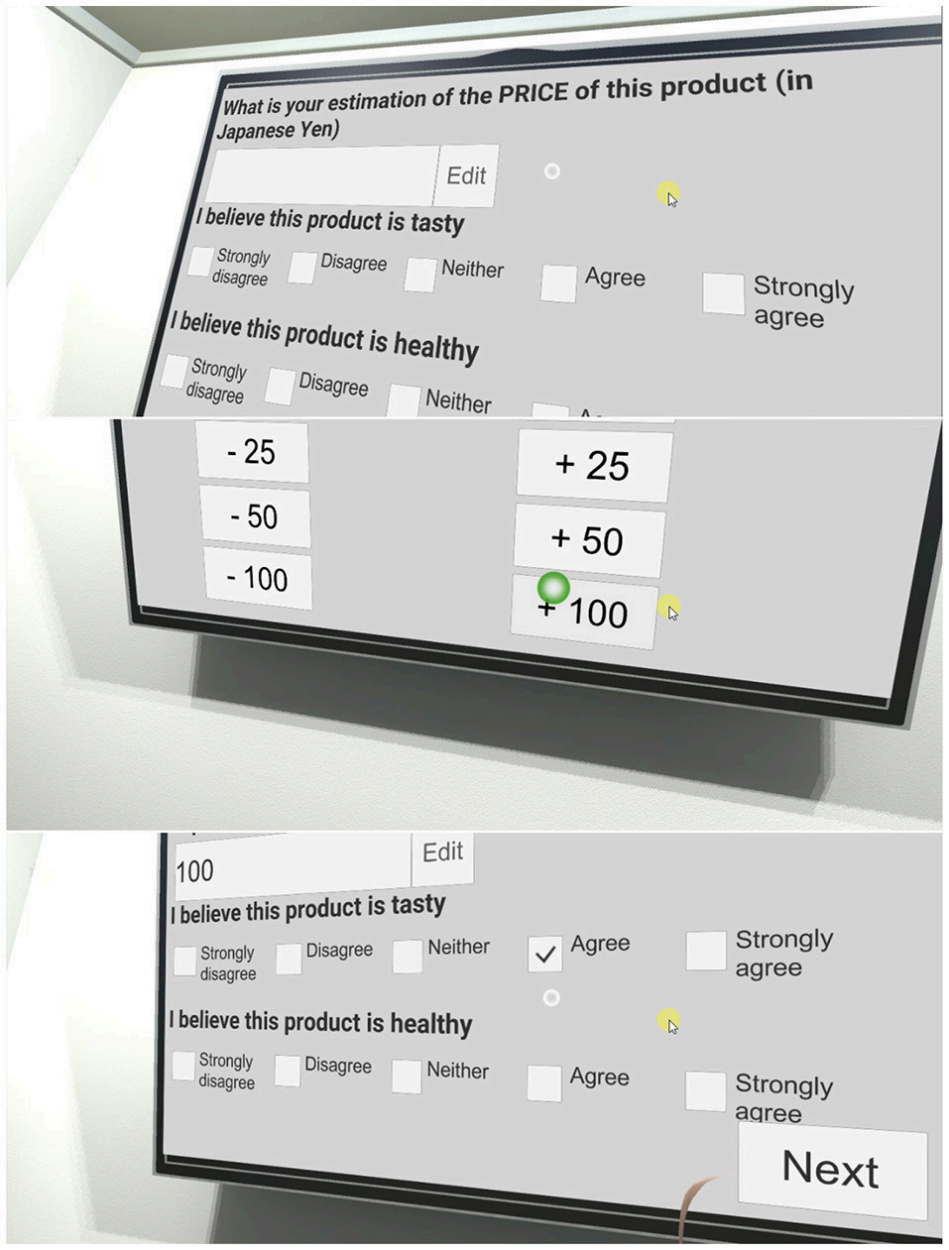
Answering questionnaires in the VE using our gaze-based interaction metaphor. **Top:** Illustration of three questions. **Middle:** Answering questions about numbers. **Bottom:** Answering Likert-scale questions.

**Figure 4:**
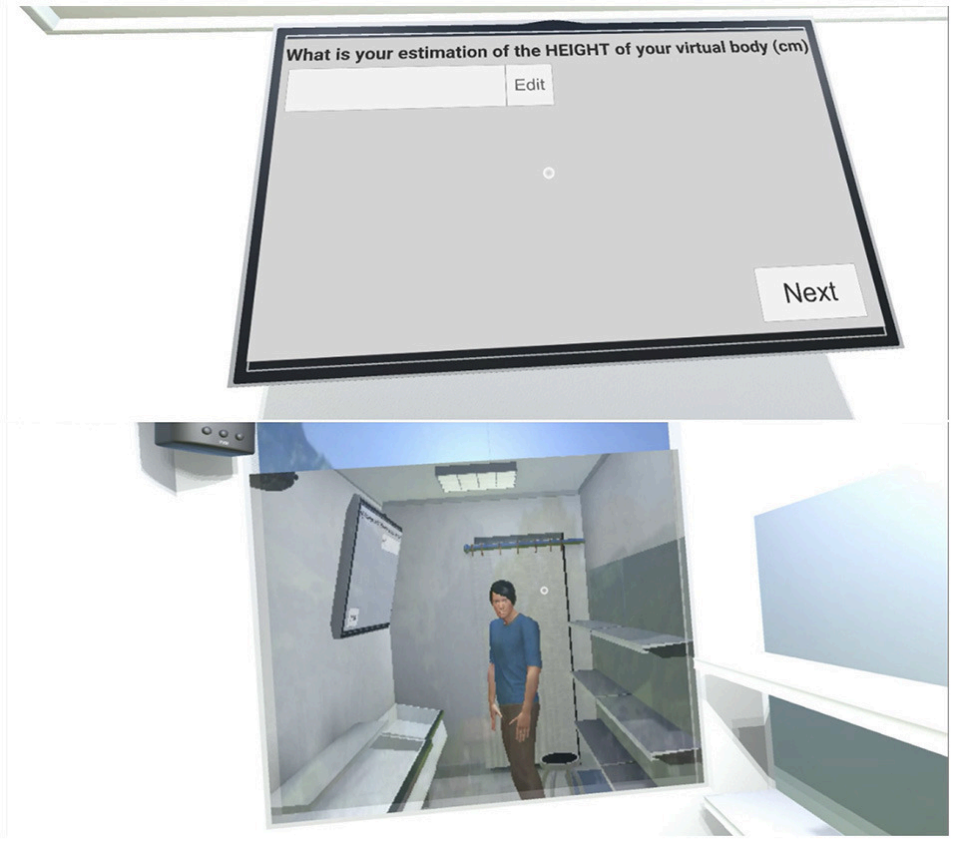
**Top:** A question about the participant's virtual body. **Bottom:** The participant decided to stand up and look at his/her reflection in the mirror before answering.

The same metaphor was chosen to fill in the questionnaires directly in the VE. As illustrated in Figure 3, we designed our questionnaires to be easily filled-in directly in the VE even when using our gaze-based metaphor. Note that we used the same design for all questionnaires that were answered directly in the VE, were they about the products (cf. Figure 3) or the virtual body (cf. Figure 4). This allowed participants to look at their avatar if they wanted to while answering questions about the virtual body (in Figure 4 the participant decided to stand up and look at his/her reflection in the mirror before answering).

### 3.3. Products

All edible products of the store were widely available in Japanese grocery stores and supermarkets. Prices were reported from an average of surrounding real stores at the time of the experiment. Almost all products were hand modeled in Blender and textured with 2D high resolution pictures of real products (cf. Figure 2). Fruits and vegetables, as well as bottles, were 3D scanned in high resolution with the Artec Eva 3D scanner^4^ since their cylindric shape prevented us from taking high quality pictures needed for texturing.

To evaluate a product's “perceived healthiness”, we calculated products' Nutrient Profile (NP) scores (using the UK Ofcom Nutrient Profiling Model, see Rayner et al., 2009). The NP score is computed with the following ingredients of the food (or drink) for 100 g of nutritional content:

- energy (*kJ*);
- saturated fat (*g*);
- total sugar (*g*);
- sodium (*mg*);
- fruits, vegetables and nuts content (% expressed as the sum of the percentages of fruits, vegetables and nuts);
- dietary fibers (*g*)^5^;
- protein (*g*).

**Figure 5:**
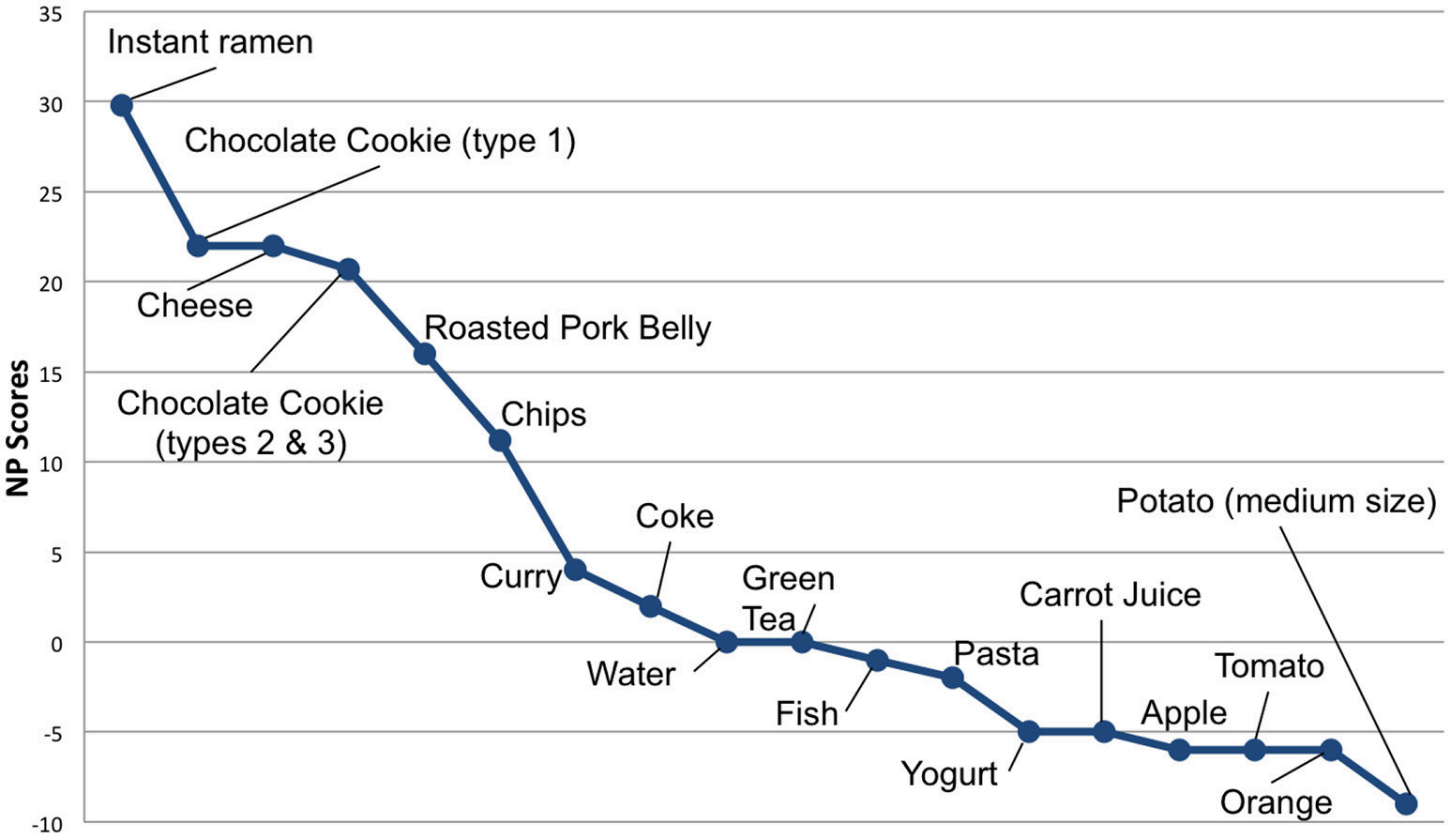
Products displayed in the virtual store along with their NP scores. The lower the score the “healthier” the product.

When the required ingredients to calculate the NP score were unavailable, we gathered them either from the producer's website (if available) or from the USDA Food Composition Databases^6^, for a highly similar product. Food products healthiness is inversely proportional to the NP score (the lower the NP score, the healthier the product). Moreover, a food (resp. drink) is deemed unhealthy with a NP score >4 (resp. >1). NP scores of our food products are presented in Figure 5.

### 3.4. Materials and embodiment

**Figure 6:**
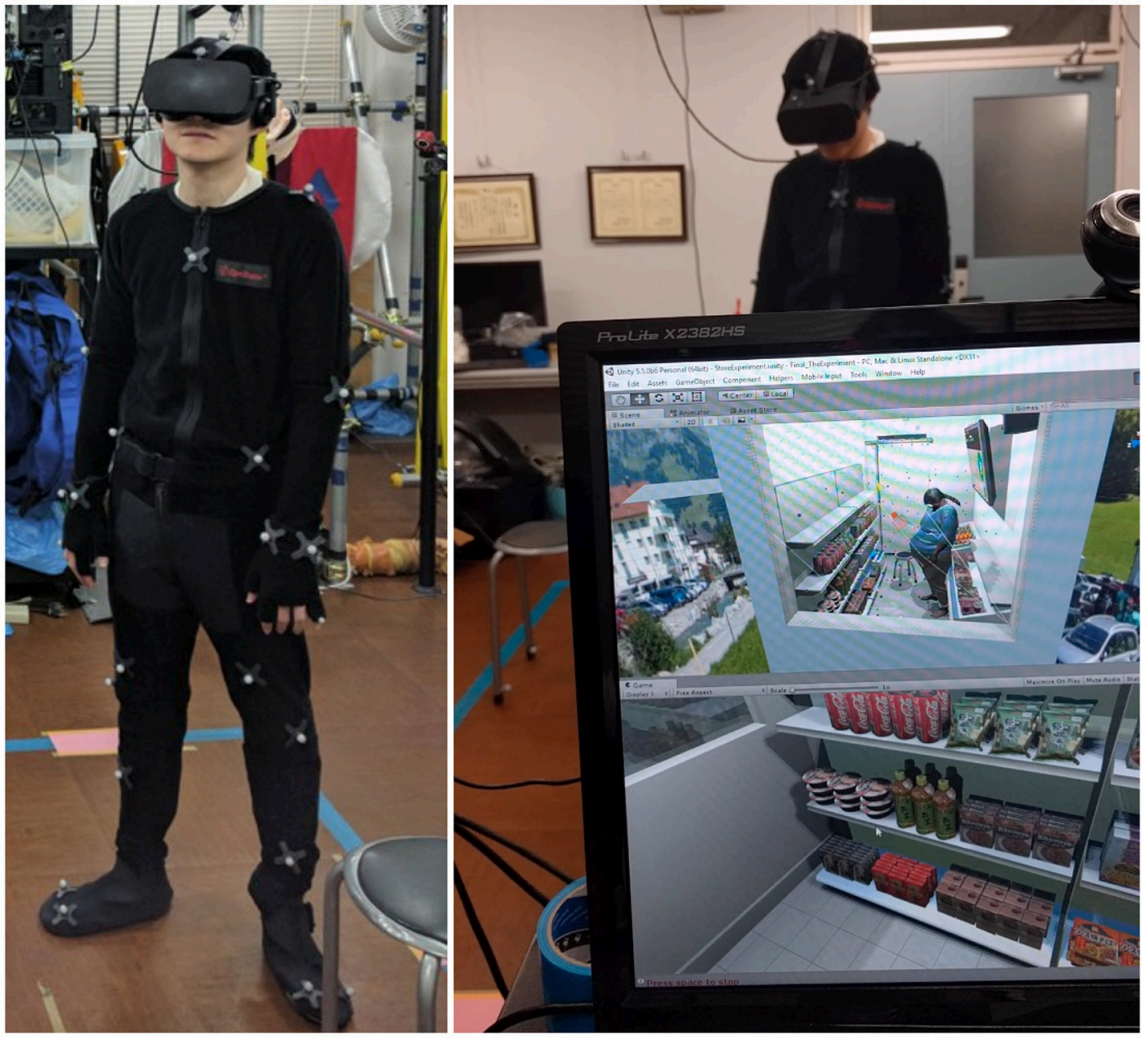
**Left:** Participant in a motion capture suit. **Right:** A participant immersed in our virtual store in an obese virtual body. On the computer screen, the top view is a third person perspective view of the virtual environment while the bottom view corresponds to the participant's point of view.

Tracking was performed by 11 Optitrack Flex 3 cameras^7^ in a space of about 6*m*^2^ (3 × 2 *m*). Participants wore an Optitrack suit matching their size with 35 markers attached (cf. Figure 6). They were immersed in the VE with an Oculus CV1^8^ HMD.

A stool was tracked with 4 markers. During the training phase, participants were sitting on the stool while for the rest of the session, they could move or sit freely. None of the participants reported any sign of motion sickness, exertion, or tracking issues.

## 4. Experiment

We present in this section the design and the protocol of the experiment.

### 4.1. Experimental design and participants

The experiment (January 2017) took place in Keio's engineering department in Japan. There were 23 participants: 21 males (age: *M* = 22.52, *SD* = 0.87) and 2 females (age: *M* = 22.5, *SD* = 0.71). The participants were all students, among which 2 (≈ 9%) had no previous experience with VR, 14 (≈ 61%) had some experience and 7 (≈ 30%) had extensive experience with VR. Participants' BMI scores were normal on average (*M* = 21.20 *kg*/*m*^2^, *SD* = 2.05, *min* = 17.37, *max* = 24.77). Two extra participants were excluded from the experiment since their BMI scores were out of the WHO “normal” BMI category (their BMI scores were respectively 27.85 and 30.47). We also excluded 4 participants that were fasting before the experiment (they were about to perform the experiment right before lunch).

We used a between-subjects design with 2 groups. The independent variable was the *weight* of the virtual body (Weight_*VB*_). Participants were randomly assigned to the control group (Group N) or to the manipulated group (Group OB):

- **N**: Virtual body with a “normal” weight according to the WHO's normal BMI category (World Health Organization, 2017a) (i.e., healthy weight). 13 participants were embodied in a virtual body with a normal BMI (≈ 23.25 *kg*/*m*^2^ for men and ≈ 19.5 *kg*/*m*^2^ for women). See Figure 1 **left**.
- **OB**: Obese virtual body. 12 participants were embodied in a virtual body with obesity (BMI ≈ 53 *kg*/*m*^2^ for men and ≈ 49.5 *kg*/*m*^2^ for women). See Figure 1 **right**.

For further details on the avatars' bodies and BMI scores, please refer to section3.1.

We computed mean and standard deviations regarding participants' BMI scores:

- **N**: *M* = 21.37 *kg*/*m*^2^, *SD* = 2.15, *min* = 17.37, *max* = 24.77;
- **OB**: *M* = 20.97 *kg*/*m*^2^, *SD* = 1.89, *min* = 18.82, *max* = 23.88.

In order to ensure data normality of our participants' BMI scores, we computed the Kolmogorov-Smirnov Z score. Results show that our data follow a normal distribution (*Z* = 0.753, *p* = 0.622).

We also computed an ANOVA to make sure both groups were comparable in terms of participants' BMI, which was the case [$F_{\left(1,\;21\right)}=0.216,p=0.647,\eta^{2}=0.010$].

### 4.2. Experimental protocol

Participants were invited one-by-one to join the experiment. Only the participant and the assistant were present in the room. Participants had to fill in a demographic questionnaire and sign a consent form. Then, they read the experimental scenario, common to both groups and put on the motion-capture suit and the HMD. Throughout the experiment, they were helped by the assistant and explicitly asked to stop if there was any sign of discomfort (such as dizziness).

Once in the VE, participants followed a two-step training session (steps I and II) before entering the virtual store (step III). Then, they went shopping according to the scenario's guidelines (see Table 1), and upon completion were asked to fill in three questionnaires (step IV). Finally, they left the VE, removed the HMD, as well as the motion-capture suit, and filled in a post-experimental questionnaire (step V). Questionnaires of steps IV and V are available as Tables 2 and 3 respectively.

**Table 1:** The experimental scenario, common to both groups. There was more than enough money to buy food for two meals.

You recently received a gift card of 5,500 Japanese Yen (¥) to spend in one of the nearby stores. You decide to go today and to buy food for your lunch and dinner (you will eat alone today). You do not have to spend everything today, and if there is nothing you would like to buy in this store, you do not have to buy anything at all!

**Table 2:** Questions on the Meal (M), the Virtual Body (VB) and the Products (Pr).

**ID**	**Question**
M1	Estimate the daily energy coverage of the chosen products (100% is enough food for 1 day)
M2	Overall, I believe the chosen products are tasty
M3	Overall, I believe the chosen products are healthy
M4	Overall, I believe the chosen products are sustainable
M5	Overall, I like the chosen products
VB1	What is your estimation of the age of your virtual body
VB2	What is your estimation of the height of your virtual body (in meters; floating point)
VB3	What is your estimation of the weight of your virtual body (in kilograms; floating point)
VB4	What is your estimation of the gender of your virtual body
VB5	What is your estimation of the geographic region of origin of your virtual body
VB6	What is your estimation of the highest academic degree received of your virtual body
VB7	What is your estimation of the profession status of your virtual body
Pr1	What is your estimation of the price of this product in Japanese Yen (¥)
Pr2	I believe this product is tasty
Pr3	I believe this product is healthy
Pr4	I believe this product is sustainable
Pr5	Overall, I like this product

**Table 3:** Questions on Embodiment (E) and Presence (P).

**ID**	**Question**
E1	I experienced that my body was located at the same position as my virtual body
E2	It seemed like I was in control of my virtual body
E3	I felt as if the virtual body was my body
E4	I felt as if my head and body were at different locations, almost as if I had been “decapitated”
E5	I felt as if my head and eyes were located at the same place as the cameras, and my body just below the cameras
E6	I experienced that I was located some distance behind the visual image of myself, almost as if I was looking at someone else
E7	It felt as if I was causing the movement I saw
E8	Whenever I moved my body I expected the virtual body to move in the same way
E9	It seemed as if the virtual body had a will of its own
E10	I felt as if I was looking at my own arms
P1	I felt like being in the virtual environment
P2	I felt like the virtual environment was like the reality
P3	I felt like in a real store

It should be noted that all interactions within the VE (i.e., with a product or for answering questionnaires) were done with a “gaze-based” interaction metaphor (cf. section 3.2.2).

### 4.3. Steps I and II - training

The training session was composed of two stages and took 5 min on average. Stage I took place in a training VE aimed at eliciting embodiment (by provoking synchronous sensory stimulations between the participant's real and virtual bodies). Stage II was designed to train participants to interact with the VE.

**Figure 7:**
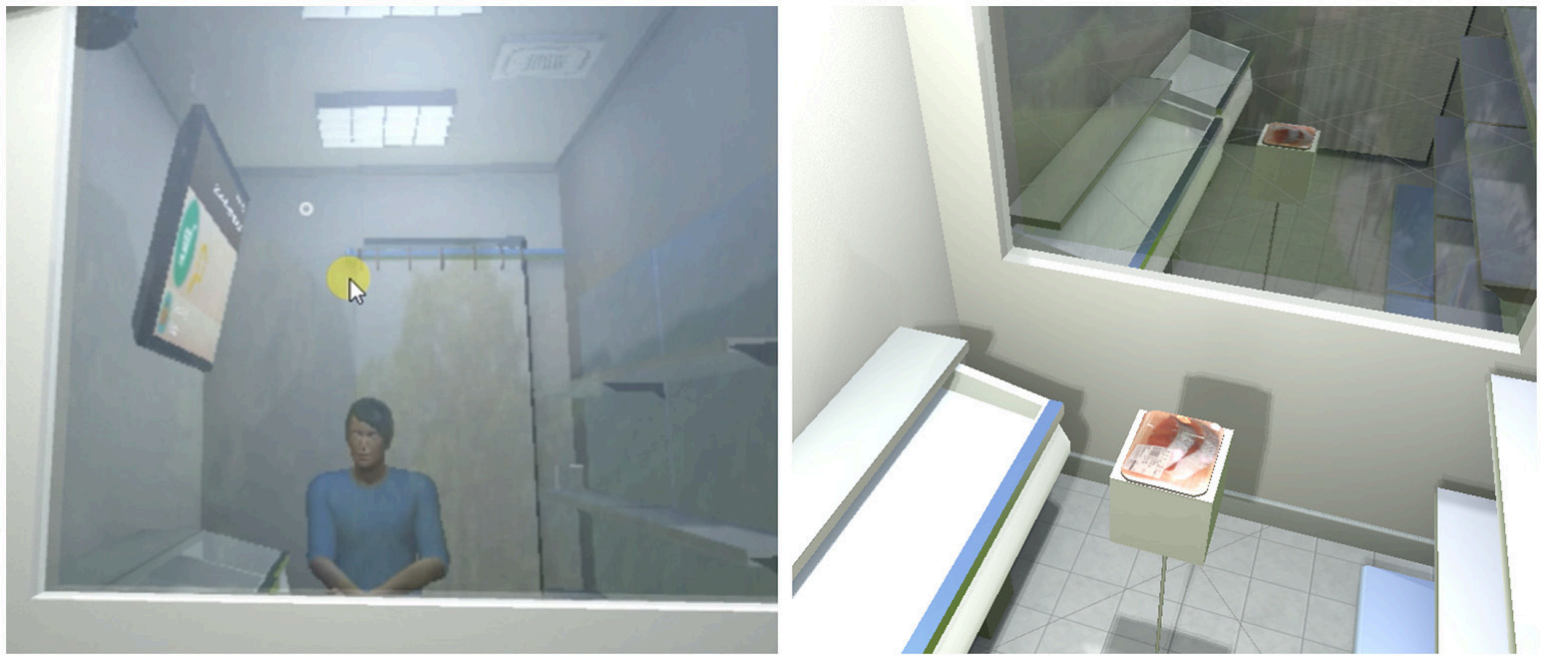
Illustrations of the training session **(left)** and of the *Product Perception* questionnaire (**right**, here evaluating Salmon slices).

**Stage I**: Participants started in front of a virtual mirror reflecting their virtual body. They were asked to move their arms and then to move closer to the mirror, to grab a tracked stool and to sit on it (cf. Figure 7 **left**). The assistant had to validate both actions to continue.

**Stage II**: The VE was replaced by the virtual store (cf. section 3.2). It was composed of a set of shelves and a big TV screen attached to a wall. At the beginning of this stage, only three small cubes were displayed on the shelves. A succession of messages on the TV asked participants to select the cubes, to add them in, and to remove them from the shopping basket.

### 4.4. Step III - shopping

Participants were then asked to shop according to the experimental scenario (i.e., shopping for two complete meals, cf. Table 1). All the products of the virtual store were displayed on the shelves (cf. Figure 2) and the participants could move freely in the shop to look at and select the products they wanted to buy.

Upon completion, participants sat on the stool to indicate the assistant they were done with the task. This procedure took 4 min on average. We logged the participants' actions (i.e., adding/removal of products) and their timings (cf. section 5.5).

### 4.5. Step IV - questionnaires in the virtual environment

All questionnaires were inspired by peer-reviewed international publications (except the *Virtual Body Perception* one, created for this paper) and translated into Japanese by a Japanese native speaker expert in user studies. The questions and the list of possible answers appeared on the TV and participants answered them by directly interacting with the TV. If the answer required a number (e.g., “What is your estimation of the age of your virtual body”), participants answered by increasing or decreasing a number on the TV. They had to fill-in three questionnaires (for which we measured the time to answer) in the VE:

- **VB**: The *Virtual Body Perception* questionnaire focused on the participants' perception of their virtual body. Since we wanted to know the virtual body's perceived age, ethnicity, education degree and employment, it was very similar to the demographic questionnaire filled-in when they entered the room. We added questions on its perceived height and weight, to calculate the avatar's perceived BMI.
- **Pr**: The *Product Perception* questionnaire [inspired by Verain et al. (2016)]. Participants had to rate (on a 5-point Likert scale, except for the estimated price [*Pr*1]) each of those products: Salmon slices; 1 Tomato; Pork belly; Instant curry; Coke^9^ (33cl); (cold) Green tea (50cl); Instant ramen; Carrot juice (50cl); 1 Apple; Chocolate cookies. The products were displayed one at a time on a shelf in the VE. In order to limit the time spent in the VE, we selected 10 products so that they cover most food categories (e.g., fruits and vegetables, instant food, soda, snack, etc.) and NP scores (cf. Figure 5).
- **M**: The *Meal Perception* questionnaire, from Bucher et al. (2015), focused on the perception of all products purchased during step III of the experiment (i.e., purchasing food for the two meals). Except for *M*1, all items were rated on a 5-point Likert scale. Upon display of the questionnaire, only the purchased products remained in the virtual store, see Figure 7 **right** and Figure 3 **bottom**.

### 4.6. Step V - questionnaires after leaving the virtual environment

After removing the HMD and the motion-capture suit, participants had to answer a post-experimental questionnaire on a desktop computer before leaving the experimental room. It was composed of two sub-questionnaires, all rated on 5-point Likert scale:

- **E**: 10 items on embodiment, inspired from several articles on embodiment in VR, namely Ehrsson (2007), Aspell et al. (2009), and Kalckert and Ehrsson (2012).
- **P**: 3 items on presence, from the Slater-Usoh-Steed Questionnaire, see Slater et al. (1994).

## 5. Results

In this section we first report statistics about our participants and group (section 5.1), before detailing how participants perceived their avatars in the immersive virtual store (section 5.2). section 5.3 is dedicated to an analysis of how participants evaluated the products proposed in our immersive virtual store. section 5.4 reports scores of the embodiment and presence questionnaires while section 5.5 investigates participants' shopping behavior.

We computed Kolmogorov-Smirnov Z values to assess data normality (see details in the Supplementary Material). Our data follow a normal distribution except for questions *M*2 (about tastiness of the products bought by participants) and *M*4 (products sustainability) of the *M* questionnaire.

Note that to compare our groups, we systematically performed analyses of variance (ANOVAs using Bonferroni corrected alpha values) using a confidence interval of 95%. As a consequence, a results is considered significant when *p* ≤ 0.05, and is represented in the following in bold.

Finally, regarding the products, it should be noted that they can be studied either in a global manner (all products together), individually and that we also decided to split them into two categories:

- **Non-healthy products**: grouping the 8 items with a NP score ≥2, cf. Figure 5. Those items are: Instant Ramen, Chocolate Cookie (type 1), Cheese, Chocolate Cookies (types 2 and 3), Roasted Pork Belly, Chips, Curry, Coke.
- **Healthy products**: grouping the 10 items with a NP score ≤ 0, cf. Figure 5. Those items are: Water, Green Tea, Fish, Pasta, Yogurt, Carrot Juice, Apple, Tomato, Orange and Potato.

Grouping our items into **non-healthy** and **healthy** products allows us to study both products perception and shopping behavior in a finer way.

### 5.1. Groups homogeneity

We tested the homogeneity of our two groups by computing Chi-squared (χ^2^) for the non-metric variable *Ethnicity*, as well as an ANOVA for the metric variable *Weight*. Both groups are homogeneous in *Ethnicity* (χ^2^ = 0.002; *df* = 1; *p* = 0.968) and *Weight* [$F_{\left(1,\;21\right)}=0.023,p=0.882,\eta^{2}=0.001$].

Overall, the participants spent 20 min in the VE and 35 min in the experimental room. The participants' oral feedback underlined a positive interest in the experiment, suggesting that they performed the experiment and answered the questionnaires seriously.

### 5.2. Avatar perception

When performing a Chi-squared test, no significant difference appeared between both groups regarding *VB* questionnaire's non-metric variables, ethnicity (*VB*5, χ^2^ = 4.301; *df* = 5; *p* = 0.507), academic degree (*VB*6, χ^2^ = 1.976; *df* = 2; *p* = 0.372) and professional status (*VB*7, χ^2^ = 4.428; *df* = 2; *p* = 0.109). Numerous studies pointed out links in developed countries between obesity and low SES, cf. Delva et al. (2006), where a higher BMI is associated to a lower education degree and employment (Tyrrell et al., 2016). Yet, participants of the OB group did not evaluate their avatars as having a lower education degree and employment, even if the avatars were clearly perceived as obese; embodiment does not seem to have conveyed those SES stereotypes.

We performed ANOVAs of the avatar's perceived: Age_*VB*_; Height_*VB*_; Weight_*VB*_ (cf. Table 4). As expected, participants of the N group perceived their avatar as weighting significantly less than those of the OB group. Participants of the OB group spent significantly more time evaluating their virtual body than participants of the N group.

**Table 4:** Mean, Standard Deviation (SD) and computed ANOVAs for the Age_*VB*_ (Estimated age of the virtual body), Height_*VB*_ (Estimated height of the virtual body, in centimeters), Weight_*VB*_ (Estimated weight of the virtual body, in kilograms), BMI_*VB*_ (Estimated BMI of the virtual body, computed using both Height_*VB*_ and Weight_*VB*_) and Time_*VB*_ (Time spent answering questions about the virtual body) variables.

**Variable**	**N (*****n*** **= 13)**		**OB (*****n*** **= 10)**		**ANOVA**		
	**Mean**	**SD**	**Mean**	**SD**	***F*_(1, 21)_**	***p***	**η^2^**
Age_*VB*_	27.0	4.56	32.30	7.63	4.307	0.490	0.170
Height_*VB*_	169.62	8.32	168.80	5.67	0.070	0.793	0.003
Weight_*VB*_	63.92	9.13	115.50	22.17	58.220	**<0.001**	0.735
BMI_*VB*_	22.14	1.93	40.68	8.12	64.008	**<0.001**	0.753
Time_*VB*_	168.40	59.94	226.91	71.98	4.529	**0.045**	0.177

### 5.3. Perception of the products proposed in our immersive virtual store

We performed ANOVAs on each item of the *Pr* questionnaire and did not detect significant difference except for the coke and the apple. Moreover, there were no significant differences in the total time taken to rate the products.

Since the *Pr* questionnaire focused on 10 products of our experiment (cf. section 4.5), we report here only significant results. For the sake of completeness, all results are available as Supplementary Material. Participants of the OB group perceived the coke as significantly healthier, and the apple as significantly tastier than those of the N group, see Table 5.

**Table 5:** Mean, Standard Deviation (SD) and computed ANOVAs of the *Product Perception* (Pr) questionnaire regarding the coke and the apple, cf. Table 2.

**Question**	**N (*****n*** **= 13)**		**OB (*****n*** **= 10)**		**ANOVA**		
	**Mean**	**SD**	**Mean**	**SD**	***F*_(1, 21)_**	***p***	**η^2^**
**Coke**							
Product healthiness on a 5-point Likert scale (*Pr*3)	1.54	0.52	1.10	0.32	5.524	**0.029**	0.208
**Apple**							
Product tastiness on a 5-point Likert scale (*Pr*2)	3.77	1.01	4.60	0.52	5.570	**0.028**	0.210

Finally, we also computed ANOVAs on the grouped Healthy and Non-Healthy products (see Table 6). There appear no significant differences (with α = 0.05) between participants of our N and OB groups in terms of perception of 10 representative products proposed in our immersive virtual store.

**Table 6:** Mean, Standard Deviation (SD) and computed ANOVAs for the Product Perception (*Pr*) questionnaire grouped by healthy (i.e., NP score ≤ 0) and non-healthy products, cf. Table 2.

**Question**	**N (*****n*** **= 13)**		**OB (*****n*** **= 10)**		**ANOVA**		
	**Mean**	**SD**	**Mean**	**SD**	***F*_(1, 21)_**	***p***	**η^2^**
**NON-HEALTHY PRODUCTS (5 REPRESENTATIVE PRODUCTS)**							
Estimated price of non-healhty products in ¥ (*Pr*1)	1139.15	234.49	1337.50	218.43	4.287	0.051	0.170
Products tastiness 5-point Likert scale (*Pr*2)	20.23	2.42	21.30	1.42	1.535	0.229	0.068
Products healthiness 5-point Likert scale (*Pr*3)	11.54	2.30	11.70	2.41	0.027	0.871	0.001
Products sustainability 5-point Likert scale (*Pr*4)	12.92	1.93	12.80	1.62	0.026	0.873	0.001
Products likeness 5-point Likert scale (*Pr*5)	18.77	2.17	19.10	2.38	0.121	0.731	0.006
**HEALTHY PRODUCTS (5 REPRESENTATIVE PRODUCTS)**							
Estimated price of healhty products in ¥ (*Pr*1)	845.08	149.36	957.80	184.79	2.623	0.120	0.111
Products tastiness 5-point Likert scale (*Pr*2)	19.77	1.42	19.90	1.91	0.035	0.852	0.002
Products healthiness 5-point Likert scale (*Pr*3)	20.69	2.69	22.40	0.97	3.637	0.070	0.148
Products sustainability 5-point Likert scale (*Pr*4)	16.23	2.39	17.60	2.63	1.702	0.206	0.075
Products likeness 5-point Likert scale (*Pr*5)	20.00	1.78	20.20	2.10	0.061	0.807	0.003

### 5.4. Embodiment and presence

The computed Cronbach's alpha coefficients for the *Embodiment* (10 items) and *Presence* (3 items) scales were respectively 0.745 and 0.619. We computed the ANOVAs on the grouped 10 Embodiment items and 3 Presence items and did not observe any significant difference (cf. Table 7). It should be noted that when studied individually, a single significant difference exists in the *Embodiment* questionnaire. This difference concerns question *E*3 (“I felt as if the virtual body was my body”) where participants of the OB group rated significantly lower than participants of the N group [*F*_(1, 21)_ = 5.237, *p* = 0.033, η^2^ = 0.200].

**Table 7:** Mean, Standard Deviation (SD) and computed ANOVAs for the *Embodiment* and *Presence* items (*P*).

**Variable**	**N (*****n*** **= 13)**		**OB (*****n*** **= 10)**		**ANOVA**		
	**Mean**	***SD***	**Mean**	***SD***	***F*_(1, 21)_**	***p***	**η^2^**
Embodiment (grouping 10 items)	37.54	6.05	34.70	7.47	1.016	0.325	0.046
*E*1	3.85	1.21	3.40	1.71	0.536	0.472	0.025
*E*2	4.31	0.21	3.60	1.26	3.101	0.093	0.129
*E*3	3.62	1.19	2.40	1.35	5.237	**0.033**	0.200
*E*4	3.92	1.19	3.80	1.23	0.059	0.811	0.003
*E*5	3.15	1.34	3.40	1.43	0.179	0.676	0.008
*E*6	3.54	1.39	3.30	1.25	0.181	0.675	0.009
*E*7	4.15	0.80	3.90	1.10	0.411	0.528	0.019
*E*8	4.15	0.80	4.50	0.71	1.167	0.292	0.053
*E*9	4.00	0.71	3.80	1.03	0.304	0.587	0.014
*E*10	2.85	1.21	3.00	1.15	0.095	0.761	0.004
Presence (grouping 3 items)	10.62	2.02	10.40	2.12	0.062	0.806	0.003
*P*1	4.46	0.52	4.50	0.53	0.031	0.863	0.001
*P*2	3.00	1.15	3.00	1.15	0.000	1.000	<0.001
*P*3	3.15	0.90	2.90	0.99	0.411	0.528	0.019

Even if our participants had an average BMI of 21.20 (resp. 21.37 and 20.97 for the N and OB groups, cf. section 4.1), there was no significant impact on the embodiment and presence items for the participants embodied in an obese avatar of much higher BMI (OB group, BMIs of the virtual avatars of ≈ 53 for men and ≈ 49.5 for women). Those results are in line with previous findings, where avatars with a different body type elicit similar embodiment and presence levels, (cf. eg., Normand et al., 2011; Kilteni et al., 2012b; Peck et al., 2013).

### 5.5. Consumer behavior data

In this section we report descriptive statistics about participants' consumer behavior (i.e., the type and number of products bought) before studying how they perceived the products they decided to chose as their meal.

#### 5.5.1. Number, type and composition of products bought

We performed ANOVAs but did not find any significant difference (cf. Table 8) between our two conditions regarding:

- the time spent in the immersive virtual store;
- the total number of products bought;
- and the number of products bought per category (healthy/non-healthy).

**Table 8:** Mean, Standard Deviation (SD) and ANOVAs for the time spent in the store, the number of products bought.

**Data**	**N (*****n*** **= 13)**		**OB (*****n*** **= 10)**		**ANOVA**		
	**Mean**	***SD***	**Mean**	***SD***	***F*_(1, 21)_**	****p****	**η^2^**
Time (s)	237.53	114.82	247.44	136.66	0.036	0.852	0.002
Total products bought	8.54	3.26	7.90	3.81	0.187	0.669	0.009
Total non-healthy Products	3.08	1.26	3.40	1.26	0.372	0.549	0.017
Instant ramen	0.54	0.52	0.70	0.67	0.423	0.523	0.020
Chocolate cookie (type 1)	0.15	0.38	0.20	0.42	0.077	0.784	0.004
Cheese	0.23	0.44	0.20	0.42	0.029	0.867	0.001
Chocolate cookie (types 2&3)	0.38	0.51	0.20	0.42	0.865	0.363	0.040
Roasted pork belly	0.62	0.51	0.60	0.52	0.005	0.944	<0.001
Chips	0.31	0.48	0.20	0.42	0.315	0.581	0.015
Curry	0.62	0.65	0.80	0.63	0.466	0.502	0.022
Coke	0.23	0.44	0.50	0.53	1.790	0.195	0.079
Total healthy products	5.46	3.15	4.50	2.92	0.561	0.462	0.026
Water	0.15	0.38	0.40	0.70	1.181	0.290	0.053
Green tea	1.00	0.82	0.40	0.52	4.109	0.056	0.164
Fish	0.23	0.60	0.40	0.52	0.507	0.484	0.024
Pasta	0.23	0.44	0.20	0.42	0.029	0.867	0.001
Yogurt	0.54	0.52	0.80	0.42	1.681	0.209	0.074
Carrot juice	0.38	0.51	0.20	0.42	0.865	0.363	0.040
Apple	0.62	0.77	0.30	0.48	1.287	0.269	0.058
Tomato	0.85	1.21	0.90	1.10	0.012	0.914	0.001
Orange	0.46	0.66	0.10	0.32	2.531	0.127	0.108
Potato	1.00	1.15	0.80	1.14	0.172	0.683	0.008

We also computed ANOVAs regarding the NP score or the composition of the products bought (cf. Table 9). Again we did not find any statistical significant difference between participants of the N and the OB groups.

**Table 9:** Mean, Standard Deviation (SD) and computed ANOVAs for the NP score and the composition of the products bought, cf. section 3.3.

**Products**	**Data**	**N (*****n*** **= 13)**		**OB (*****n*** **= 10)**		**ANOVA**		
		**Mean**	***SD***	**Mean**	***SD***	***F*_(1, 21)_**	****p****	**η^2^**
Total	NP score	22.84	23.63	29.04	25.73	0.360	0.555	0.017
	Energy (*kJ*)	6361.64	2343.32	6136.50	3244.90	0.037	0.848	0.002
	Sat. Fat (*g*)	29.73	13.32	27.97	18.17	0.072	0.791	0.003
	Sugar (*g*)	66.87	30.36	67.92	35.69	0.006	0.940	<0.001
	Sodium (*mg*)	2058.58	1127.88	2531.37	1775.67	0.608	0.444	0.028
	NSP Fibers (*g*)	11.61	5.13	8.74	5.74	1.597	0.220	0.071
	Protein (*g*)	40.22	22.90	44.11	19.26	0.186	0.670	0.009
Non-healthy	NP score	48.69	19.24	49.84	27.51	0.014	0.907	0.001
	Energy (*kJ*)	4962.75	1993.62	4826.05	2824.10	0.019	0.893	0.001
	Sat. Fat (*g*)	29.10	13.22	26.89	18.42	0.112	0.741	0.005
	Sugar (*g*)	48.64	28.66	56.71	32.64	0.397	0.535	0.019
	Sodium (*mg*)	1986.62	1160.51	2452.34	1790.76	0.572	0.458	0.027
	NSP Fibers (*g*)	5.23	3.23	4.56	3.45	0.233	0.634	0.011
	Protein (*g*)	24.43	11.36	23.16	15.84	0.050	0.825	0.002
Healthy	NP score	−25.85	16.88	−20.80	14.61	0.566	0.460	0.026
	Energy (*kJ*)	1398.89	1302.99	1310.45	1231.67	0.027	0.870	0.001
	Sat. Fat (*g*)	0.63	1.50	1.08	1.29	0.569	0.459	0.026
	Sugar (*g*)	18.24	14.93	11.21	5.44	1.991	0.173	0.087
	Sodium (*mg*)	71.97	68.99	79.02	43.40	0.080	0.780	0.004
	NSP Fibers (*g*)	6.37	4.67	4.18	3.75	1.471	0.239	0.065
	Protein (*g*)	15.79	17.45	20.95	14.83	0.561	0.462	0.026

Overall we can say that there is no significant difference in shopping behavior between our two groups. In regard to our hypothesis HH1, we can conclude that, given our data, **there is no statistical evidence** (with α = 0.05) that people embodied in obese avatars follow the classical stereotypes associated with obese people food purchases (i.e., buying more unhealthy food products and less healthy food products).

#### 5.5.2. Meal perception

We performed ANOVAs on each item of the *M* questionnaire and on the time spent to answer the questionnaire (*Time*_*M*_) but we did not detect any significant difference (cf. Table 10).

**Table 10:** Mean, Standard Deviation (SD), computed ANOVAs and time taken (Time_*M*_) for the meal (*M*) questionnaire, cf. Table 2.

**Question**	**N (*****n*** **= 13)**		**OB (*****n*** **= 10)**		**ANOVA**		
	**Mean**	**SD**	**Mean**	**SD**	***F*_(1, 21)_**	***p***	**η^2^**
Estimated % of daily energy coverage (*M*1)	74.62	23.05	61.93	19.72	1.935	0.179	0.084
Products tastiness 5-point Likert scale (*M*2)	4.15	0.55	4.22	0.42	0.093	0.764	0.004
Products healthiness 5-point Likert scale (*M*3)	2.85	0.80	2.38	1.06	1.452	0.242	0.065
Products sustainability 5-point Likert scale (*M*4)	2.69	0.63	2.88	0.57	0.537	0.472	0.025
Products likeness 5-point Likert scale (*M*5)	4.38	0.65	4.32	0.67	0.052	0.822	0.002
Time_*M*_	101.28	35.52	99.94	11.54	0.013	0.910	0.001

In regard to our hypothesis HH2, given our data, we can infer that **there is no statistical evidence** (with α = 0.05) that people embodied in obese avatars find non-healthy food products as less unhealthy than people embodied in non obese avatars.

## 6. Discussion

While we did not detect deep changes in customer purchase behavior and food products perception, our study raises interesting questions.

Indeed, while participants of the OB group did not perform the shopping task in accordance to our expectation (i.e., following the negative *weight stigma* stereotype by buying more products and more unhealthy or high energy intake products), neither did they buy significantly more healthy products than the N group. If it were the case, it could imply that participants of the OB group wanted to transform their virtual bodies back into their real bodies by eating more healthy food (i.e., trying to improve their health).

The same remark also holds regarding food products perception: while participants of the OB group did not perceive unhealthy or high energy intake products as less unhealthy neither did they find healthy products more healthy than participants of the N group.

Both observations tend us to believe that stereotype activation failed for our participants in two possible ways: while the *weight stigma* stereotype was not elicited, neither was its contrary (i.e., participants' trying to improve the health of their obese avatar by buying more healthy products).

In the following, we present and discuss possible explanations (PE) of this absence of stereotype activation for our participants.

### 6.1. Avatar perception and embodiment

While previous findings showed that embodied participants tend to act as their virtual body hints them to, we did not obtain such results. This may be due to a concerning limitation of our experiment: the too high contrast between the participants' real and virtual bodies. As suggested by Yoo et al. (2015), this may have weakened the activation of the construct associated with obese people, therefore provoking less effects from changes in perception and behavior.

A first potential confirmation could be seen in the significant difference noticed in question *E*3 (“I felt as if the virtual body was my body”) where participants of the OB group rated significantly lower than participants of the N group, see Table 7.

This could be further supported by informal feedback we obtained from our participants at the end of the experiment. Indeed, two participants from the OB group told us that they “did not like being in an obese avatar”. One of them further stressed that he lacked space in the immersive virtual store. Finally another participant of the OB group told us that he “did not feel associated at all with the avatar”.

This leads us to suggest a possible explanation 1 (PE1):

- **PE1**: Embodiment avatar's body dependence: it is possible that not all avatars' body representations induce behavioral changes. When embodiment was elicited, behavioral changes have been observed to depend on the type of body, however unlike body types previously studied an obese virtual body holds deep stigmatizing stereotypes. It is possible that behavioral changes cannot be expressed in a body that the participant rejects or did not feel as his/her own.

Nevertheless, a natural question arises: Why did we not, unlike previous research, detect behavioral modifications of our OB participants? See e.g., Kilteni et al. (2013) on how participants play music depending on their avatars, Banakou et al. (2013) on embodiment in children virtual bodies or Peck et al. (2013) on dark skinned avatars.

We assume that a major difference between our virtual body and previous embodiment research is that they focus only on visual changes (too some extent for Banakou et al., 2013 who used children virtual bodies). We hypothesize that an obese virtual body may require some other non-visual stimulus, e.g., the sensation of the extra weight or the change in body size. This may be manipulated for example by adding a cushion to our obese participants.

Even if the OB group did not need more time to adapt to a different body type in the training session, and rated presence and embodiment comparably to the N group, it took them significantly longer to examine and rate their avatar. This can be seen as another sign of the difficulty for OB participants to really be embodied into obese avatars. They probably needed much more time to rate a body that is so different from their own.

### 6.2. Shopping behavior

Previous work showed a negative correlation between products bought and thus money spent in (real) supermarkets and BMI, see Lear et al. (2013). Unlike them, we did not detect any significant difference in our immersive virtual supermarket. This could obviously be due to the slightly different buying processes in virtual and real stores we mentioned previously.

However, we performed additional statistical tests in order to understand our participants' behavior during the shopping task in the immersive virtual store. In order to do so, we computed correlations and regressions between shopping data and different aspects of our groups of participants.

First of all, we investigated the relationship between participants' BMI and their shopping behavior (number of products bought). We thus computed Pearson's correlations between participants BMI and the number of products bought. Results (cf. Table 11) showed that for the **OB condition**, there is no correlation between participants BMI and: (i) the total number, (ii) the number of non-healthy and (iii) the number of healthy products bought. When studying results for individual products, the only significant correlation that exists for the OB condition is a positive correlation between participants' BMI and the number of cheese bought (*r* = 0.678, **p = 0.031**). Regarding the **N condition** (cf. Table 11) there is a significant and positive correlation between the participants BMI and the number of products bought (*r* = 0.656, **p = 0.015**). This leads us to investigate further this correlation by studying separately the healthy and the non-healthy products bought. Results (cf. Table 11) show that there is a significant and positive correlation between the participants BMI and the number of healthy products bought, *r* = 0.731, **p = 0.005**. This means that for the N group participants with a higher BMI bought more healthy products. However, no such significant correlation exists for the total number of non-healthy products bought.

**Table 11:** Pearson correlation between participants' BMI for each condition and the number of products bought.

**Data**	**N (*****n*** **= 13)**		**OB (*****n*** **= 10)**	
	***r***	***p***	***r***	***p***
Total products bought	0.656	**0.015**	0.573	0.083
Total non-healthy products	−0.133	0.664	0.449	0.193
Instant ramen	−0.576	**0.039**	0.054	0.883
Chocolate cookie (type 1)	0.471	0.105	0.018	0.961
Cheese	0.342	0.252	0.678	**0.031**
Chocolate cookie (types 2&3)	−0.054	0.861	−0.434	0.211
Roasted pork belly	0.150	0.625	0.113	0.755
Chips	−0.538	0.058	−0.135	0.709
Curry	−0.127	0.680	0.362	0.304
Coke	0.222	0.465	0.362	0.303
Total healthy products	0.731	**0.005**	0.555	0.096
Water	0.205	0.501	0.608	0.062
Green tea	−0.340	0.256	0.088	0.810
Fish	0.606	**0.028**	0.141	0.698
Pasta	0.342	0.252	0.032	0.931
Yogurt	−0.213	0.486	0.404	0.247
Carrot juice	0.379	0.202	0.017	0.963
Apple	0.502	0.080	0.330	0.352
Tomato	0.628	**0.022**	0.207	0.566
Orange	0.643	**0.018**	−0.178	0.623
Potato	0.292	0.333	0.487	0.153

Looking at the products individually, there are four significant correlations for the N group between the participants' BMI and the number of some of the products bought, namely:

- a negative correlation for the instant ramen (*r* = −0.576, **p = 0.039**);
- a positive correlation for the number of fish (*r* = 0.606, **p = 0.028**);
- a positive correlation for the number of tomatoes (*r* = 0.628, **p = 0.022**);
- a positive correlation for the number of oranges (*r* = 0.643, **p = 0.018**).

Then, we investigated the degree to which the condition (N or OB) could predict shopping behavior. To this end we computed two logistic regressions. Table 12 shows the logistic regression coefficient, Wald test and statistical significance of the influence of condition (N or OB) on participants' consumer behavior. Results show that, with α = 0.05, the condition of the experiment does not predict the total number of products bought. In the same vein, the condition of the experiment does not predict the number of non-healthy products and the number of healthy products bought by the participants.

**Table 12:** Binary Logistic Regressions predicting the number of total products, the number of healthy and of non-healthy products bought from condition (N or OB).

	**Total products**				
**Predictor**	**B**	**Wald χ**^2^	***p***			
Condition	−0.057	0.202	0.653			
	**Non-healthy products**			**Healthy products**		
**Predictor**	**B**	**Wald χ**^2^	***p***	**B**	**Wald χ**^2^	***p***
Condition	0.283	0.597	0.440	−0.142	0.750	0.386

Finally, we studied the degree to which ratings of the Embodiment and Presence questionnaires (cf. Table 7) could predict shopping behavior. This could tell us whether participants who rated significantly higher in embodiment and/or presence had a different shopping behavior. Three linear regressions were calculated per condition (N and OB) to predict: (i) the total number of products bought, (ii) the number of healthy products and (iii) the number of non-healthy products based on the scores of the embodiment and presence questionnaires, cf. Table 13. Results show that, for the two conditions studied (N and OB), neither Embodiment (group N: *p* = 0.673, group OB: *p* = 0.438) nor Presence (group N: *p* = 0.594, group OB: *p* = 0.105) were significant predictors of the number of products bought. The same non significant results were found for Embodiment and Presence regarding non-healthy (resp. Embodiment, group N: *p* = 0.449, group OB: *p* = 0.664; and Presence, group N: *p* = 0.381, group OB: *p* = 0.187) and healthy (resp. Embodiment, group N: *p* = 0.455, group OB: *p* = 0.241; and Presence, group N: *p* = 0.364, group OB: *p* = 0.112) products.

**Table 13:** Three linear Regressions predicting per condition (N or OB) the number of (i) total products (ii) non-healthy products and (iii) healthy products bought based on the embodiment and presence scores.

		**Total products**			**Non-healthy products**			**Healthy products**		
**Predictor**		**Standardized coefficients B**	***t***	***p***	**Standardized coefficients B**	***t***	***p***	**Standardized coefficients B**	***t***	***p***
N	Constant		1.388	0.195		1.305	0.221		0.953	0.363
	Embodiment	0.227	0.435	0.673	−0.401	−0.798	0.449	0.394	0.777	0.455
	Presence	−0.287	−0.550	0.594	0.465	0.916	0.381	−0.482	−0.951	0.364
OB	Constant		2.345	0.051		1.599	0.154		2.393	0.048
	Embodiment	−0.281	−0.822	0.438	0.154	0.454	0.664	−0.434	−1.280	0.241
	Presence	−0.637	−1.864	0.105	−0.498	−1.464	0.187	−0.617	−1.818	0.112

From these statistical analyses (regressions and correlations), we can conclude, given α = 0.05, that:

- the group of participants (N or OB) cannot predict their shopping behavior;
- the scores of embodiment and presence do not predict shopping behavior, neither in terms of number nor in type (non-healthy or healthy) of products bought by our participants;
- there is no significant correlation between our participants' BMI and their shopping behavior, except for the number of healthy products and thus the total number of products bought for participants of the N condition.

Additionally, we propose a possible explanation 2 (PE2) to the absence of significant difference in terms of shopping behavior between our N and OB conditions:

- **PE2**: Higher-level cognitive processes leading to a modification of behavior might require something more than embodiment and presence. Kilteni et al. (2013) suggested that the embodiment drive behavioral changes when the virtual body is more appropriate to do a task than the “real one”. It is therefore possible that people may have ignored their virtual body for the shopping task.

One can argue that participants did not have enough contact with obese people and did not know how to shop to match the shopping behavior of obese people [only 3.7% of the Japanese population is obese, cf. OECD (2017)], but 32% of our participants were also European, where there is a larger ratio of obese people [roughly 20%, see World Health Organization (2017b)].

This could also be due to a possible explanation 3 (PE3):

- **PE3**: People with obesity have a more complex shopping behavior than anticipated that do not follow classical stereotypes (i.e., buying more products and products with more high energy intake).

### 6.3. Meal and products purchasing behavior vs. perception

There were very few significant differences when buying food products when embodied. Maybe participants of the OB group did not feel like obese people (which was supported by some informal feedback from some of our participants) even when embodied in obese avatars. Again, it might have been caused by being embodied inside a stigmatizing type of body.

Our results also point out that participants embodied in an obese avatar do not perceive non-healthy food products as healthier than people embodied in non-obese avatars. Participants of the OB group only perceived coke as significantly less healthier and apple as significantly tastier. Even if both results could be seen as hints that participants of the OB group experienced a shift of perception, we did not detect any statistical evidence supporting HH2.

This cognitive dissonance (i.e., the lack of systematical links between consumers' buying behavior and perception), seems very weak, if existing at all. Indeed, we did not find any other perception differences neither regarding products with high calories content (such as chocolate cookies, instant ramen, etc.) nor regarding healthy products such as fruits and vegetables.

Both remarks tend to give more credit to our PE2.

## 7. Limitations

There are several limitations to the present study. Our sample size (*n* = 23) was limited, and we were unable to test other hypotheses, such as an overweight avatar instead of an obese one. Moreover, our participant pool (college students in Japan) was not very diverse, and participants with a different background and culture could have yielded different results. Those are however common issues of this type of experiments.

### 7.1. Using BMI as an indicator of obesity

Still related to the country where our experiment was carried out, our decision to use BMI for the indication of a “non-overweight and healthy” or “overweight and unhealthy” virtual body can be challenged. Indeed, a WHO expert consultation (World Health Organization Expert Consultation, 2004) has shown that the mean or median BMI for Asian populations is lower than that observed for non-Asian populations (and hence the BMI distribution is shifted to the left). Nevertheless, the same expert consultation (World Health Organization Expert Consultation, 2004) also showed that the tendency toward abdominal obesity might be greater in Asian than in non-Asian populations.

As a consequence, there is a debate concerning the values of the cut-off points used in the BMI categories (World Health Organization, 2017a) since they may underestimate obesity-related risks in these populations. The WHO expert consultation (World Health Organization Expert Consultation, 2004) concluded that, although the mean or median BMI for Asian populations is lower than that observed for non-Asian populations, Asians generally have a higher percentage of body fat than white people of the same age and sex. As a consequence, BMI cut-off values should probably be modified for different populations (in particular they should be lowered for Asian populations).

Still related to the use of BMI in our study, there is an on-going debate (Zhao et al., 2013) regarding the validity of using BMI as an indicator of obesity and of predicting the percentage of body fat (PBF). Indeed, some studies suggested that the Body Adiposity Index (BAI), which is computed as: hip circumference (*cm*) / stature (*m*)^1.5^; should be used as an alternative to BMI.

Finally, the Waist-to-Height ratio (WHtR), also called Waist-to-stature ratio (WSR), is another indicator that can be used. For example, some studies showed that WHtR is better than BMI at predicting life expectancy (see Ashwell et al., 2014), as well as cardiovascular risks (see e.g., Lee et al., 2008; Schneider et al., 2010). Nevertheless, in the particular case of Japan, it was shown that WhtR was not superior to BMI for predicting cardiometabolic risks (see Hori et al., 2014).

In this study, we were not interested in using obese avatars to raise concerns of our participants regarding health issues such as life expectancy or cardiovascular risks. We wanted our participants to realize they were embodied in visually obese avatars in order to see if it could affect their perception of food products and consumer behavior. As a consequence and given our objectives, it is not clear whether BMI was the best indicator to use or if we should have relied on other indicators such as WHtR, waist circumference (WC) or BAI. Nevertheless, our purpose was not to study BMI or to evaluate it as an indicator. Our aim was that our participants could identify themselves as obese in the virtual world without any doubt. We chose to rely on BMI to qualify our virtual body but we could have used any other indicator since we only aimed at producing virtual avatars that would be recognized as obese without any doubt.

### 7.2. Interaction mechanism

We embodied the participants in a virtual body, and immersed them in a virtual store. We are confident that both the embodiment and immersion were, from a VR point-of-view, sufficient. However, immersion in marketing science requires more than a 3D virtual store. For edible products, the experience of appropriation (see Hansen and Mossberg, 2013), is especially important and go through several steps, where subjects build their desire for a product from internal and external stimuli. Because of the constraints of our experiment, we did not include any appropriation steps for the participants to inquire about what they wanted to purchase, but rather “put them” in the VE.

One might wonder why we chose to use a “gaze-based” metaphor when interacting with the virtual store when our participants were full-body tracked. We see two possible alternatives: using a controller or gestures.

While using a controller would have been easy, it could have hindered participants in their movements and would not have felt more natural than our solution. Nevertheless, using controllers could have been a more natural and intuitive selection mechanism than our proposal. Indeed, grabbing products with hand-held controllers would have forced participants to see their virtual arms more often than with our solution. Nevertheless, hand-held controllers, by favoring manual interaction with products, could also have reduced the sense of presence in participants since collisions with 3D objects were not handled and no haptic feedback was implemented.

We could also have implemented gestures to pick objects but decided not to use this solution since: (1) we did not have finger tracking and the gesture might have felt unrealistic to users (ours is also very unrealistic); (2) we felt the lack of haptic feedback would have disturbed the participants (3) this may have caused unrealistic collisions with the virtual objects (a hand going through a product or a shelf). We consciously chose the “gaze-based” technique because we thought gestures would not be better and would even be more of a distraction than an intuitive interaction mechanism.

## 8. Conclusion

In this paper we studied participants' perception and purchase behavior of food products in a virtual store when embodied in a normal or an obese virtual body. We only noted a change of perception for the obese group where the coke was perceived as healthier and the apple as tastier. While this hints that modifying the weight of avatars could impact participants' perception of food products, those results were nevertheless not linked to a change of purchase behavior regarding food products, as suggested by the additional analyses performed (correlations and regressions). As future work, we would like to embody obese participants in non-obese avatars to validate our findings. As mentioned before, using an obese avatar with a lower BMI could also give us a better understanding of our current results. Another interesting question related to embodiment and product perception and purchase behavior would be to study whether or not a virtual body is needed in the context or virtual supermarkets. Would having a virtual body or not modify participants behavior and perception?

Unlike prior work on embodiment, which suggested that avatars influence participants' behavior, we did not notice such modifications. We hypothesized that this may be due to our special type of obese avatars, which, unlike previous work, may require more than just visual modifications for participants to really embody in this new virtual body. Indeed, an obese body modifies not only the visual aspect of an avatar but has more profound changes (extra weight and body size, etc.) and would likely require additional stimuli for the participant on top of the visual one. This could be backed up by our results showing that participants embodied in virtual avatars spent significantly more time when evaluating their virtual avatars (about their height, weight and age). Finally, the absence of stereotype activation might be due to its “negative” nature, i.e., participants rejected their virtual bodies while performing the task. This could mean that stereotype activation might be more complex that previously reported in the literature (e.g., Bailenson and Ahn, 2011; Peña et al., 2012; Kilteni et al., 2013; Yoo et al., 2015).

In order to confirm this hypothesis some further experiments need to be carried out to study whether adding some external cues such as cushions around the waist to participants embodied in an obese virtual avatar would significantly modify ratings in terms of embodiment and presence. A careful study of the motion of participants embodied in an obese avatar would also be very interesting. Does they unconsciously change the way they move? As guidelines for researchers studying embodiment, we believe it is very important to try to have participants notice as earlier as possible and as often as possible during the experiment their virtual body, by adding mirrors, having them move around the VE, etc. This is obviously true for any study on embodiment but we believe it is even more crucial when the virtual body differs greatly form the real one. Obviously this raises questions about the ecological validity of the excitement (e.g., putting mirrors within a virtual store would certainly seem awkward and implausible).

The reasons why participants perceived some of the products differently remain beyond the scope of this study. Food behavior is an on-going and complex research area, where food choice is linked to hedonic, ecological, utilitarian, symbolic and personal preferences.

This research was, to the best of our knowledge, the first use of virtual embodiment to study shopping behavior and products perception in a virtual store. Given our results and the future research questions we proposed, it remains unclear what are the impacts of having a virtual body on purchase behavior and products perception. We believe that it would be very interesting to study if virtual avatars should be as close as possible to the participants' bodies (in terms of hair and skin color, body type, etc.) or if it would not make substantial differences when trying to activate behavioral stereotypes. Finally, regarding stereotype activation and virtual embodiment, our results tend to show that the impact on participants' behavior might be more nuanced, especially with strong negative stereotypes such as weight stigma, than previously reported in the literature.

## Ethics statement

Since this study was non-invasive, a formal revision and approval by Keio University Research Ethics Committee was not necessary. Nevertheless, the study was carried out in accordance with the recommendations of the guidelines of the Keio University Research Ethics Committee. All subjects gave written informed consent in accordance with the Declaration of Helsinki.

## Data availability

The raw data supporting the conclusions of this manuscript will be made available by the authors, without undue reservation, to any qualified researcher.

## Author contributions

AV, J-MN and CL conceived and designed the experiment with the help of GM and MS. The computer programming was carried out by AV. The experimental questionnaires were translated from English to Japanese by MS. The experiment was carried out by AV. Data analysis was carried out by CL, with the help of J-MN. The paper was written by J-MN, CL, and AV with the help of all the authors, and reviewed by all the authors.

### Conflict of interest statement

The authors declare that the research was conducted in the absence of any commercial or financial relationships that could be construed as a potential conflict of interest. The reviewer, SH, and handling editor declared their shared affiliation.

## Footnotes

^1^http://www.manuelbastioni.com/manuellab.php

^2^http://www.marvelousdesigner.com/

^3^https://unity3d.com/

^4^https://www.artec3d.com/3d-scanner/artec-eva

^5^Using the non-starch polysaccharides (NSP) method.

^6^https://ndb.nal.usda.gov/ndb/

^7^http://optitrack.com/products/flex-3/

^8^https://www.oculus.com/rift/

^9^In this paper we use the term *coke* to represent a cola-like soft drink.
